# The role of PSMA-based radioligand therapy in hormone-sensitive prostate cancer

**DOI:** 10.1007/s00259-025-07083-8

**Published:** 2025-02-12

**Authors:** Riccardo Laudicella, Matteo Bauckneht, Irene A Burger, Alberto Cacciola, Stefano Fanti, Andrea Farolfi, Vincenzo Ficarra, Andrei Iagaru, Virginia Liberini, Stefano Pergolizzi, Giulia Santo, Irene Virgolini, Fabio Minutoli, Sergio Baldari

**Affiliations:** 1 Nuclear Medicine, Department of Biomedical and Dental Sciences and Morpho-Functional Imaging, University of Messina, Messina, Italy; 2 IRCCS Ospedale Policlinico San Martino, Genova, Italy; 3 Nuclear Medicine, Department of Health Sciences (DISSAL), University of Genova, Genova, Italy; 4 Department of Nuclear Medicine, University Hospital Zürich, University of Zurich, Zurich, Switzerland; 5 Department of Nuclear Medicine, Cantonal Hospital Baden, affiliated Hospital for Research and Teaching, University of Zurich, Baden, Switzerland; 6 Brain Mapping Lab, Department of Biomedical, Dental Sciences and Morphological and Functional Imaging, University of Messina, Messina, Italy; 7 Nuclear Medicine Division, IRCCS Azienda Ospedaliero-Universitaria di Bologna, Policlinico S. Orsola, Bologna, Italy; 8 Nuclear Medicine, Alma Mater Studiorum, University of Bologna, Bologna, Italy; 9 Gaetano Barresi Department of Human and Paediatric Pathology, Urologic Section, University of Messina, Messina, Italy; 10 Division of Nuclear Medicine and Molecular Imaging, Department of Radiology, Stanford University, Stanford, USA; 11 Nuclear Medicine Unit, ASO S.Croce e Carle Cuneo, Cuneo, Italy; 12 Radiation Oncology Unit, Department of Biomedical, Dental and Morphological and Functional Imaging Sciences, University of Messina, Messina, Italy; 13 Department of Nuclear Medicine, Medical University of Innsbruck, Innsbruck, Austria; 14 Department of Experimental and Clinical Medicine, ’’Magna Graecia’’ University of Catanzaro, Catanzaro, Italy

**Keywords:** HSPC, PCa, Prostate-specific membrane antigen, RLT, Theranostics

## Abstract

**Purpose:**

Conventional systemic therapies are valuable options in prostate cancer (PCa); however, such treatments can determine adverse events and toxicity. The observed improvement in overall survival, coupled with PSA reduction and a favorable safety profile in the post-taxane castration-resistant PCa (CRPC) setting has prompted the consideration of PSMA-based radioligand therapy (RLT) earlier in the treatment sequence. In this review, we will describe the literature and ongoing clinical trials regarding the use of PSMA-based RLT in hormone-sensitive PCa (HSPC) including the neoadjuvant, de-novo/synchronous metastatic, adjuvant, and early BCR settings.

**Methods:**

We performed a systematic literature search on the PubMed/MEDLINE/EMBASE and clinicaltrials.gov databases for studies and protocols assessing the role of PSMA-based RLT in HSPC.

**Results:**

The literature search yielded 140 results. After screening titles and abstracts and applying inclusion and exclusion criteria, we selected 25 papers showing the potentialities of earlier RLT in HSPC, with several ongoing trials.

**Conclusion:**

Early use of PSMA-based RLT holds significant potential in HSPC patients from the neoadjuvant to the BCR setting. In these stages, the lower tumor burden, more frequent exclusive nodal involvement, and higher organ reserve may improve treatment efficacy and allow for treatment combinations while maintaining a less toxic profile.

## Introduction

Prostate-specific membrane antigen (PSMA) is overexpressed in nearly 90% of prostate cancer (PCa), especially in later-stage metastatic castration disease (mCRPC) [1–3].

**Fig. 1:**
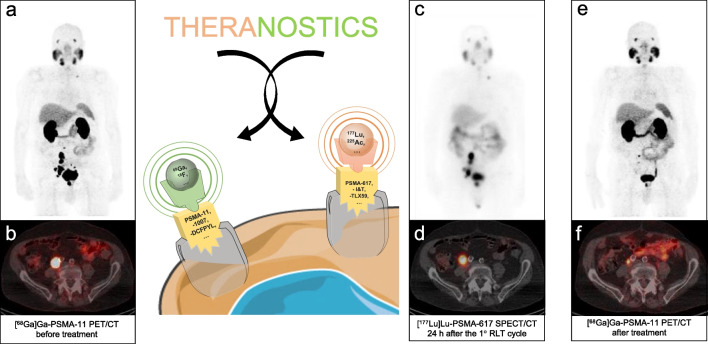
Schematic representation of the theranostics concept through a case example. The term “theranostics” is derived from the combination of “therapy” and “diagnosis”, summarizing the concept of using a specific molecule (i.e., PSMA) to first identify areas of high target expression with a diagnostic agent (e.g., [^68^Ga]Ga or [^18^F]), and subsequently treat them with a therapeutical agent (e.g., [^177^Lu]Lu or [^225^Ac]Ac) for effective treatment. We present the case of an 80-year-old patient diagnosed with metastatic prostate adenocarcinoma (ISUP 4, Gleason Score 4 + 4) in 1995 initially treated with RT followed by hormone therapy. By 2016, the patient had become castration-resistant and underwent therapy with abiraterone, followed by enzalutamide. Due to disease progression and refused chemotherapy, he underwent RLT with [^177^Lu]Lu-PSMA-617 in early 2017. Enrollment [^68^Ga]Ga-PSMA-11 PET/CT (**1a**, maximum intensity projection - MIP;** 1b**, fused axial images) showed high PSMA expression in the prostatic bed and lymph node metastases. The patient underwent four cycles of [^177^Lu]Lu-PSMA-617 RLT, with a cumulative administered activity of 24.9 GBq. Post-treatment planar whole-body images (**1c**, anterior acquisition) acquired 24 h post-injection after the first RLT confirmed a high radiopharmaceutical concentration in the lesions previously identified by [^68^Ga]Ga-PSMA-11 PSMA PET/CT as further magnified by [^177^Lu]Lu-PSMA-617 SPECT/CT (**1d**, fused axial images). The patient showed a progressive decline in PSA values (from 76.9 ng/ml to 0.8 ng/ml) and exhibited a partial response to 4 RLT cycles at post-treatment [^68^Ga]Ga-PSMA-11 PET/CT (**1e**, MIP; **1f**, fused axial images)

In this scenario, radioligand therapy (RLT) with [^177^Lu]Lu-PSMA offers an effective therapeutical option for patients with high PSMA expression at positron emission tomography (PET) [4]. RLT is the perfect representation of the theranostics approach where a specific molecule (i.e., PSMA ligand) is first bound with a diagnostic nuclide (i.e., [^68^Ga]Ga or [^18^F] for PET) enabling the in-vivo and non-invasive detection of a selected pathology (i.e., PCa). If high expression/avidity is observed, the same/equivalent molecule will be bound to a therapeutical nuclide (i.e., the beta minus emitter [^177^Lu]Lu or the alpha emitter [^225^Ac]Ac) for effective treatment. With [^177^Lu]Lu it is furthermore possible to assess the biodistribution of the RLT exploiting a minor emission quote of the already administered radiopharmaceutical (i.e., the ~ 10% of [^177^Lu]Lu γ-emission), aligning with the principle “treat what you see” for macroscopic disease [5, 6], as represented in Fig. 1.

In the first randomized phase II study, Hofman et al. involved 200 mCRPC patients previously treated with androgen-receptor pathway inhibitor (ARPI) and docetaxel. Study participants were included in the case of [^68^Ga]Ga-PSMA-11 PET-avid disease with maximum standardized uptake value (SUV_max_) > 20 at one site and > 10 at any site, without fluorodeoxyglucose (FDG)-positive/PSMA-negative localizations. Indeed, [^177^Lu]Lu-PSMA-617 was significantly superior in improving progression-free survival (PFS) and reducing PSA than cabazitaxel, together with fewer G3-4 adverse events [7]. Long-term follow-up demonstrated improved quality of life (QoL) and confirmed the reduced toxicity for RLT versus cabazitaxel; however, the authors observed a similar improvement in terms of overall survival (OS) for [^177^Lu]Lu-PSMA-617 (19.1 m) and cabazitaxel (19.6 m), describing the RLT as an “alternative to cabazitaxel” [8]. In the phase III randomized trial Vision, Sartor et al. included 831 mCRPC patients in progression after 2 or more ARPI and 1 or 2 taxane lines, with [^68^Ga]Ga-PSMA-11 PET-positive lesion/s (uptake > liver) without any measurable PSMA PET-negative localizations (lymph node with short axis ≥ 2.5 cm, solid-organ lesions/bone lesions with soft-tissue component with short axis ≥ 1.0 cm). Indeed, they demonstrated the superior efficacy of [^177^Lu]Lu-PSMA-617 plus standard of care (SoC: ARPI, steroids, radiation therapy – RT) over the SoC alone in terms of both PFS (8.7 vs. 3.4 m) and OS (15.3 vs. 11.3 m). In the Vision trial, Sartor et al. reported a higher incidence of G3 or higher-grade adverse events for the [^177^Lu]Lu-PSMA-617 + SoC cohort (52 vs. 38%) compared to the SoC cohort. However, despite this increase, the adverse events did not significantly impact the QoL [9]. As a result, [^177^Lu]Lu-PSMA-617 RLT has received FDA/EMA approval and is widely reimbursed in post-taxane advanced mCRPC [4]; also, [^177^Lu]Lu-PSMA-617 RLT demonstrated early positive results (prolonged radiographic PFS - rPFS) in PSMA PET-positive taxane-naïve mCRPC patients in comparison to ARPI-change, despite no OS improvement which can be partially explained by a high portion of crossover patients from the ARPI-change arm (134/234, 57%) [10].

In PCa patients, several effective systemic therapy options are available prior to RLT: the best established are hormone- and chemotherapy, but for selected patients also immune- or targeted therapy might be an option. However, these treatments can lead to substantial adverse events and toxicities.

Conventional (androgen-deprivation therapy – ADT) and next-generation hormone therapy (ARPI) are approved (also in combination) as adjuvant therapies [11] as well as more advanced disease stages [12]. Nonetheless, both ADT and ARPI can determine weight gain, sexual dysfunction, rash, fatigue, cognitive impairment, and hypertension [13–15].

Chemotherapy with docetaxel represents a valuable treatment option for patients with de-novo metastatic PCa, also in association with ADT, as well as for more advanced stages of the disease. In addition, cabazitaxel is approved for PCa patients who have progressed after or during docetaxel-based chemotherapy [12]. However, docetaxel and cabazitaxel can result in collateral effects such as neutropenia and anemia [7, 16, 17].

Given these challenges, the literature provides initial evidence supporting the earlier use of [^177^Lu]Lu-PSMA-617 RLT in selected patients who are either unfit for or have refused hormone therapy/chemotherapy.

The rationale for the early use of PSMA-targeted RLT lies partly in the mechanisms underlying PSMA expression. Indeed, the gene encoding PSMA is the folate hydrolase 1 (FOLH1) which is androgen-repressed [18]; castration-resistant prostate cancer is potentially more radioresistant than HSPC due to persistent androgen receptor signaling, which leads to the upregulation of AR-regulated DNA repair genes. Consequently, AR inhibition—more pronounced in HSPC—enhances PSMA expression, supporting the potential for early application of PSMA-based RLT in PCa [19]. Beyond the influence of signaling pathways, it is well-known that PSMA expression becomes increasingly heterogeneous with disease progression and treatment pressure, with 15–20% of mCRPC completely losing PSMA expression [20].

Also, a recent survey study conducted across 95 theranostic centers worldwide revealed that 51% of the centers were already treating hormone-sensitive patients with [^177^Lu]Lu-PSMA RLT in 2022 [21].

The aim of the present work is to systematically review data on the early application of PSMA-based RLT in hormone-sensitive PCa (HSPC) patients, covering the use of this therapy from the neoadjuvant setting to biochemical recurrence (BCR) and metastatic disease before the later stage, also providing an overview of the main ongoing clinical trials in this area.

### Methods

This review was performed following the Preferred Reporting Items for Systematic Reviews and Meta-Analyses (PRISMA) statement [22]. We performed a systematic literature search on the PubMed/MEDLINE and EMBASE databases for studies and published protocols assessing the role of PSMA-based RLT in HSPC. The search algorithm included the following terms: (“PSMA” OR “Prostate-Specific Membrane Antigen”) AND (“HSPC” OR “hormone-sensitive prostate cancer”) AND (“RLT” OR “radioligand therapy”). The search was extended until October 1st, 2024, including the clinicaltrials.gov database for ongoing studies. We included original papers and case reports considering the novelty scenario. The following exclusion criteria were applied: (1) systematic review and metanalysis; (2) clinical setting other than HSPC or mixed cohorts; (3) non-English language; (4) preclinical studies; (5) editorials. Additionally, references were screened to identify other relevant papers. Five authors (RL, MB, AF, VL and GS) independently screened titles and abstracts of papers obtained after the literature search and assessed their eligibility. In the case of discrepancies between the authors, a second round of screening was performed, and any disagreement was solved by majority voting. For each selected paper, we collected the following information: (1) clinical setting; (2) study design (prospective vs. retrospective); (3) number of included participants/patients; (4) radiopharmaceutical(s) employed including timing and dosage; (5) imaging modality (PET, PET/CT, or PET/MRI); (6) study design and status (for ongoing trials); (7) main endpoints and results.

## Results

**Fig. 2:**
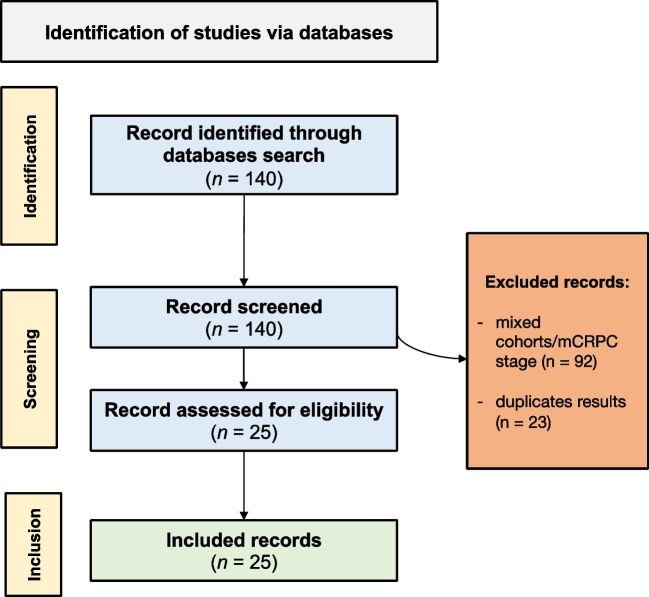
Overview of the studies selection process

The literature search yielded 140 results. After screening titles and abstracts and applying inclusion and exclusion criteria, we selected 25 papers. The principal reasons for exclusion were as follows: the presence of a heterogeneous cohorts/mCRPC scenario, and duplicate results. A flowchart summarizing the selection process is shown in Fig. 2.

### Neoadjuvant

In the neoadjuvant setting, Golan et al. assessed the safety and early outcome of 2–3 cycles of 7.4 GBq of [^177^Lu]Lu-PSMA-I&T at 2 weeks intervals before radical prostatectomy (RPE, 4 weeks after the last RLT) in 14 high-risk localized PCa with high PSMA uptake at [^68^Ga]Ga-PSMA-I&T PET/computed tomography (CT). The authors registered a PSA median reduction of 17% and 34% after 2 and 3 [^177^Lu]Lu-PSMA-I&T doses, respectively. They also observed ISUP downgrading in three patients (23%), without affecting continence recovery [23].

More recently, in the LuTectomy study, Eapen et al. investigated the dosimetry, safety, and efficacy of neoadjuvant [^177^Lu]Lu-PSMA-617 in 20 men with high-risk localized PCa with high-tumor uptake at [^68^Ga]Ga-PSMA-11 PET/CT before RPE. Study participants received one (10/20) or two cycles (6 weeks interval) of 5 GBq of [^177^Lu]Lu-PSMA-617, 6 weeks before RPE. After cycle one, the median highest tumor radiation absorbed dose for all lesions was 35.5 Gy (19.6 Gy to the prostate), and five study participants received radiation to lymph nodes. 9/20 (45%) patients achieved > 50% PSA decline without any G3 or higher toxicities. Interestingly, one patient had minimal residual disease on final histology, and no patients achieved a complete pathological response [24].

The NEPI trial is a single-center (Germany) randomized phase I/II study (not yet recruiting) that aims to explore neoadjuvant [^177^Lu]Lu-PSMA-617 with or without Ipilimumab in 58 high-risk PCa candidates for RPE (tissue specimen must be available), at maximum oligometastatic on PSMA PET with prostatic SUV_max_ > 12 (M1 patients at CI will be excluded). The study will start with a safety cohort assessing [^177^Lu]Lu-PSMA-617 dose including 6 to 12 patients (phase I); then, 46 study participants will be randomized (1:1) to receive 2 cycles of 7.4 GBq [^177^Lu]Lu-PSMA-617 (at 6 weeks interval) with or without 4 cycles of concomitant Ipilimumab 3 mg/kg every 3 weeks before RPE (day 1 RLT, day 3 Ipilimumab); ADT will be applied to all patients during the neoadjuvant treatment phase. The primary endpoints will be the feasibility of performing prostatectomy on time and the pathological complete response; the estimated study completion is September 2028 (NCT06388369).

The randomized, recruiting, phase I/II Nautilius is a single-center (US) trial evaluating 2 cycles of [^177^Lu]Lu-rhPSMA-10.1 with and without Degarelix (ADT) in 36 high-risk, localized and locoregional (M1 on PSMA PET are allowed) PSMA PET-positive (prostatic SUV_max_ > 8) PCa, candidate for RPE. Included participants must be M0 on CI and must have a minimum prostatic volume of 1.5 cm^3^ on MRI. The primary endpoints will be the safety, and the RLT efficacy based on radiation dose to the tumor and PSA assessment; the estimated study completion is June 2026 (NCT06066437).

Finally, NCT06259123 is a single-center phase II trial (Austria), recruiting 10 oligometastatic PCa planned for RPE diagnosed by [^68^Ga]Ga-PSMA-11 PET (M1a, and/or M1b with ≤ 5 bone metastases, and/or M1c with ≤ 3 lung metastases). Study participants will receive two cycles of neoadjuvant 5 GBq [^177^Lu]Lu-PSMA-I&T at 6-week intervals before RPE. The primary endpoints are safety and tolerability, with an estimated study completion date of June 2027.

These studies collectively aim to evaluate the efficacy, safety, and feasibility of [^177^Lu]Lu-PSMA RLT in the neoadjuvant setting, potentially improving outcomes for patients with high-risk prostate cancer.

### De-novo/synchronous metastatic PCa

PSMA RLT may also find space in another challenging scenario of patients with de-novo metastatic PCa ineligible for SoC treatments as initially shown by Satapathy et al. in a single patient [25], and then in a pilot study in 10 de-novo/synchronous high-volume mHSPC (9/10 > 20 lesions, mainly bone) ineligible for chemo/ARPI due to cardiometabolic disorders or unwilling. Indeed, the authors observed that a maximum of 6 cycles of [^177^Lu]Lu-PSMA-617 plus ADT reached undetectable PSA in 50% with a median rPFS of 24 months, without any G3 or higher adverse events [26].

Further insights are anticipated from the UpFrontPSMA study, which investigated the sequencing of ADT plus 2 cycles of 7.5 GBq of [^177^Lu]Lu-PSMA-617 followed 6 weeks later by docetaxel (75 mg/m^2^every 3 weeks iv for 6 cycles) versus ADT plus docetaxel (75 mg/m^2^ every 3 weeks iv for 6 cycles) in 122 study participants with de-novo high volume metastatic hormone naïve disease, where [^68^Ga]Ga-PSMA-11 PET-expression prevailed over [^18^F]FDG PET-uptake. As primary endpoint, 25/61 (41%) patients in the [^177^Lu]Lu-PSMA-617 plus docetaxel group had undetectable PSA at 48 weeks compared with 10/61 (16%) patients in the docetaxel alone group (OR 3.9, *p* = 0.002), without significantly different toxicities; furthermore, after a median follow-up of 2.5 years, the reached PSA-based PFS was 30 months in the RLT arm versus 21 months in the docetaxel alone arm (HR 0.6, *p* = 0.04) [27].

Sathekge et al. described the use of [^225^Ac]Ac-PSMA-617 in a retrospective cohort of 21 mHSPC (M1b and M1c) patients who refused conventional treatments. They used a de-escalating dosage approach with an initial administered activity of 8 MBq reduced to 7, 6, or 4 MBq based on clinical, biochemical, and imaging response ([^68^Ga]Ga-PSMA-11 PET/CT) after each cycle. After a median of 3 RLT cycles (range 2–6) performed every 8 weeks, 20/21 patients (95%) had any decline in PSA and 18/21 (86%) showed a PSA decline of ≥ 50% (including 4 patients in whom PSA became undetectable), in the absence of any G3 or higher adverse events. A lower percentage decrease in PSA following treatment was associated with increased mortality and shorter progression-free survival [28].

### Adjuvant

In the adjuvant scenario, the multicentric PROQURE dose-escalation study aims to explore the combination of SoC external beam radiation therapy (EBRT, 7 weeks), ADT (3 years) plus a single cycle of 3, 6 or 9 GBq of [^177^Lu]Lu-PSMA-617 (2nd EBRT week) in 18 PSMA PET-positive N1M0 PCa. The main endpoints will be tolerability and safety [29].

### BCR HSPC

Tulipan et al. described the case of a 64-year-old patient with International Society of Urological Pathology (ISUP) grade 4 HSPC initially treated with RT and ADT, achieving a PSA nadir of 0.3 ng/ml. During the 2nd ARPI line (apalutamide after bicalutamide), the patient complained of muscle cramps, myalgia, and kidney failure, leading to the discontinuation of apalutamide and the start of a watchful waiting approach. At the time of BCR (PSA of 2.8 ng/ml), the patient underwent a [^18^F]PSMA-1007 PET/contrast-enhanced CT (ceCT) indicative of two highly avid lumbar-aortic lymph nodes (SUV_max_ 28.5 and 8.8). The patient refused chemotherapy and received a single cycle of 8.5 GBq of [^177^Lu]Lu-PSMA-617, resulting in a BCR-FS at 22 months after RLT [30].

In another case, Demirkol et al. reported data from a young ISUP 3 HSPC patient (56 years old) initially treated with brachytherapy (PSA nadir 1.4 ng/ml). At the time of BCR (PSA 4.4 ng/ml), a PSMA PET/magnetic resonance imaging (MRI) demonstrated the presence of highly avid local recurrence in the right prostatic lobe. However, due to medical (previous major abdominal surgery for rectal cancer), physical (obesity), and social factors (young and recently married), the patient was considered ineligible for/refused other treatments, thus receiving a single cycle of 5.8 GBq of [^177^Lu]Lu-PSMA-I&T followed by a BCR-Free Survival of 24 months [31].

In a pilot study, Privè et al. prospectively assessed 10 BCR participants after local therapy, considered with low-tumor burden at [^68^Ga]Ga-PSMA-11 PET/ceCT (≤ 10 positive lesions). All patients were treated with 2 cycles of [^177^Lu]Lu-PSMA-617 at 3.7 + 3 or 6 GBq according to dosimetry and eventual toxicity after the 1st cycle. Indeed, each cycle included single-photon emission computed tomography (SPECT)/CT at 1, 24, 48, 72, 168 h post-RLT (3D dosimetry following MIRD scheme), along with blood sampling at 5, 30 min, 1, 2, 3, 24, 48, 72, 168 h post-RLT for blood dosimetry. Regarding primary endpoints, at 6 months follow-up (FU), the authors did not observe any G3 or higher adverse events. Threshold radiation doses for organs at risk (OAR) were reached, while all target lesions received therapeutic radiation doses. Specifically, the salivary glands, kidneys, and bone marrow received a median total organ absorbed dose of 3.4 Gy [1.2–5.9], 4.3 Gy [3.1–6.1], and 0.15 Gy [0.1–0.2], respectively, compared to a median dose of 12.7 Gy [4.6–48.7] to target lesions. Among secondary endpoints, the authors described a PSA 50% reduction in 5/10 (1 undetectable), stability in 2/10, and 6/10 patients were PSMA PET responders. The entire cohort postponed the use of ADT while maintaining a good QoL [32].

The same patient population was analyzed in a dosimetric fashion by Peters et al., confirming that despite a dosimetric uncertainty of ~ 25% in lesions < 1 cm, the absorbed dose and organ kinetics of [^177^Lu]Lu-PSMA-617 in low-tumor burden mHSPC were comparable to those in high-volume mCRPC [33]. Interestingly, they observed that the “sink effect” [34], briefly described as the potential increased radiation dose in OAR especially in low-volume disease, is negligible and does not increase in later treatment cycles. Namely, the organ absorbed dose was similar or lower following the II RLT cycle, and a total [^177^Lu]Lu-PSMA-617 activity of at least 38 GBq is safe. Specifically, the mean organ absorbed dose resulted in 0.39 ± 0.17 Gy/GBq to salivary glands, 0.49 ± 0.11 Gy/GBq to kidneys, 0.09 ± 0.01 Gy/GBq to the liver, and 0.017 ± 0.008 Gy/GBq to the bone marrow. They also confirmed that soft tissue lesions responded significantly better to RLT than bone, as confirmed by volumetric and PSA change [33].

The same group from the Netherlands later published a retrospective study including 20 BCR patients who had undergone primary treatment and ADT but were ineligible for/refused SoC conventional treatment due to unacceptable side effects. Of these patients, 14/20 had oligometastatic/low tumor burden disease (≤ 5 mts on [^68^Ga]Ga-PSMA-11/[^18^F]PSMA-1007 PET), while 6/20 had high-volume disease; lymph node and bone metastases were present in 20/20 and 6/20, respectively. 18/20 patients underwent 1–4 cycles of [^177^Lu]Lu-PSMA-617, while 2/20 received 1–4 cycles of [^177^Lu]Lu-PSMA-617/I&T plus at least 2 cycles of [^225^Ac]Ac-PSMA-617/I&T (“tandem” patients). Considering a median FU of 20 months, the authors reported a median PFS of 12 m (10 m for [^177^Lu]Lu-PSMA-617-only, 19 m mean PFS for tandem patients) with only one relevant toxicity consisting of G3 anemia and G4 thrombocytopenia in 1 tandem patient treated with 4 cycles of [^177^Lu]Lu-PSMA-617/I&T plus 2 cycles of [^225^Ac]Ac-PSMA-I&T. Furthermore, there were 17/20 PSA responding patients, with 7/20 PSA reduction > 90%, including the two tandem patients [35].

In another prospective pilot study, Grkovski et al. included six oligometastatic (max 3 lesions) BCR PCa after primary therapy, who underwent 2 cycles of [^177^Lu]Lu-PSMA-617 (7.5 GBq each) and stereotactic body RT (SBRT) after 6 weeks (27 Gy in 3 fractions). In each patient, [^68^Ga]Ga-PSMA-11 or [^18^F]DCFPyL PET/CT was performed at baseline, interim (after RLT), and post SBRT, while SPECT/CT was acquired at 3, 24, and 88 h after the first RLT for dosimetric analysis. As primary endpoints, the authors did not observe any relevant toxicity within at least 4 weeks after both cycles, and RLT contribution to the mean and maximum biologically effective dose (BED) was 34 and 40%, respectively [36].

### BCR HSPC recruiting/ongoing studies

#### Single arm

The role of RLT in early BCR PCa after RPE or RT is being evaluated in a single-centre/arm study from Vienna University. Indeed, the NCT06220188 is a phase II study recruiting 20 participants with PSMA PET-negative radio-morphological local recurrence (distant metastases are allowed; cN0, cM0/1) with PSA doubling time (PSA_dt_) < 12 months and Eastern Cooperative Oncology Group Performance Status (ECOG PS) 1–2. Study participants will receive 2 cycles of [^177^Lu]Lu-PSMA-I&T at 6-week intervals (3 + 6 GBq). Primary endpoints are PSA response > 50% (PSA50) and toxicity.

Furthermore, the ProstACT TARGET is a single-arm, phase II study exploring the radiolabeled PSMA-targeting antibody TLX591 ([^177^Lu]Lu-DOTA-rosopatamab) in combination with EBRT in BCR oligometastatic HSPC after definitive treatment (ISUP > 3 at histopathology). Overall, the study aims to enrol 50 participants across multiple clinical sites in Australia who will receive 2 cycles of 2.8 GBq of [^177^Lu]Lu-DOTA-TLX591, given 14 days apart. Inclusion criteria are the absence of prostatic-bed recurrence and the presence of ≤ 5 PSMA PET-positive pelvic lymph nodes. Specifically, participants must have at least one pelvic nodal lesion ≥ 5 mm in the greatest dimension with an SUV_max_ > 9 in enlarged nodes and an SUV_max_ > 3 in nodes < 5 mm. The primary outcome measure will be the PSA-FS, defined as the time from enrolment to PSA level increase of > 25% (NCT05146973).

Also, a phase I study will assess the safety (primary endpoint), feasibility and best dose of consolidative [^177^Lu]Lu-rhPSMA-10.1 after rhPSMA-7.3 (flotufolastat) PET-guided EBRT (no M1 patients) in 10 BCR patients after-prostatectomy. As secondary endpoints, patients will also undergo serial SPECT/CT and blood sample collection assessing the circulating tumor cell and dosimetry (NCT06105918).

#### Combination/comparison with SoC

PSMAddition is an ongoing multicentric, international (19 countries) phase III study comparing (1:1) SoC (ADT and ARPI) plus 6 cycles of 7.4 GBq of [^177^Lu]Lu-PSMA-617 vs. SoC in untreated/minimally treated mHSPC with positive [^68^Ga]Ga-PSMA-11 PET/CT. The study aims to enrol 1,126 participants, who will be stratified based on several factors, including disease volume, age, and previous or planned treatment of the primary tumor. Crossover is allowed, and the primary endpoint is rPFS, with OS as a key secondary endpoint [37].

Another study of association with SoC is the phase II BULLSEYE by Privè et al., which is a prospective study recruiting 58 BCR oligometastatic HSPC after primary treatment with ≤ 5 bone/lymph node lesions at [^18^F]PSMA-1007 PET/CT and whole-body MRI (wbMRI) with a PSA_dt_ ≤ 6 m. Study participants will be randomized (1:1) to receive either 2 cycles of 7.4 GBq of [^177^Lu]Lu-PSMA-617 every 6 weeks (plus 2 more cycles in still PSMA-avid patients at interim [^18^F]PSMA-1007 PET/CT) or the SoC represented by watchful wait until the initiation of ADT. Crossover to the RLT group is allowed at the end of treatment in case of clinical progression or a 100% increase in PSA from baseline. The primary endpoint is rPFS based on conventional criteria [38].

#### Combination/comparison with RT

POPSTAR II is an Australian phase II study recruiting 92 BCR oligometastatic PCa with positive [^68^Ga]Ga-PSMA-11 or [^18^F]DCFPyL PET after primary treatment. Study participants will be included if they have a maximum of 5 nodal/bone lesions with PSMA RADS 4–5 (low/absent PSMA uptake is allowed) with at least 1 lesion with PSMA SUV_max_ twice that of liver uptake. Over 24 months, study participants will be randomized (1:1) to receive either SBRT (1–3 fractions to all sites) plus 2 cycles of [^177^Lu]Lu-PSMA-617/I&T or SBRT (1–3 fractions to all sites), considering PSA-FS as the primary endpoint (NCT05560659).

PSMA-DC is another ambitious multicentric phase III study recruiting 450 BCR oligometastatic HSPC with positive [^68^Ga]Ga-PSMA-11 or [^18^F]DCFPyL PET after primary treatment. Unlike POPSTAR II, patients must have negative conventional imaging (CI, namely CT and bone scan - BS) for M1 disease (CI can be positive only for local recurrence) with at least 1 PSMA PET-positive CI-negative distant metastases without liver/brain involvement. Study participants will be then randomized (1:1) to SBRT plus 4 cycles of 7.4 GBq [^177^Lu]Lu-PSMA-617 versus SBRT alone. The primary endpoint will be the metastases-FS (RECIST1.1), while the secondary key endpoint will be the time to ADT (NCT05939414).

The LUNAR study is an ongoing monocentric study from UCLA enrolling 100 oligorecurrent HSPC. Study participants may have a maximum of 5 asymptomatic lesions outside the prostate/prostatic bed with increased radiotracer uptake on [^68^Ga]Ga-PSMA-11 PET/CT, with the smallest diameter ≥ 1 cm on CT or MRI or increased uptake on BS. Participants will be randomized (1:1) according to several factors towards 2 cycles of 6.8 GBq [^177^Lu]Lu-PSMA-I&T plus dose-adapted SBRT (based on post-RLT PSMA PET) versus SBRT to all PSMA-positive sites. The primary endpoint will be the composite PFS (PSMA PET, PSA, therapy change, death), while secondary endpoints also foresee a radiomics analysis [39].

A more complex published protocol regards the ongoing study ROADSTER, which will include (phase I *n* = 12, phase II *n* = 30) biopsy-confirmed intraprostatic recurrent PCa after RT with PSMA PET/MRI SUV_max_ ≥3 in the absence of any extraprostatic disease. Study participants will be randomized (1:1) to 1 cycle of 6.8 GBq of [^177^Lu]Lu-PSMA-I&T plus 1 high-dose-rate (HDR) brachytherapy with boost (transrectal ultrasound- and PSMA PET/MRI-guided) versus 2 HDR brachytherapy with boost. After treatment, all patients will receive a re-biopsy of the prostate for a translational study, and the primary endpoints will be safety and feasibility [40].

**Fig. 3:**
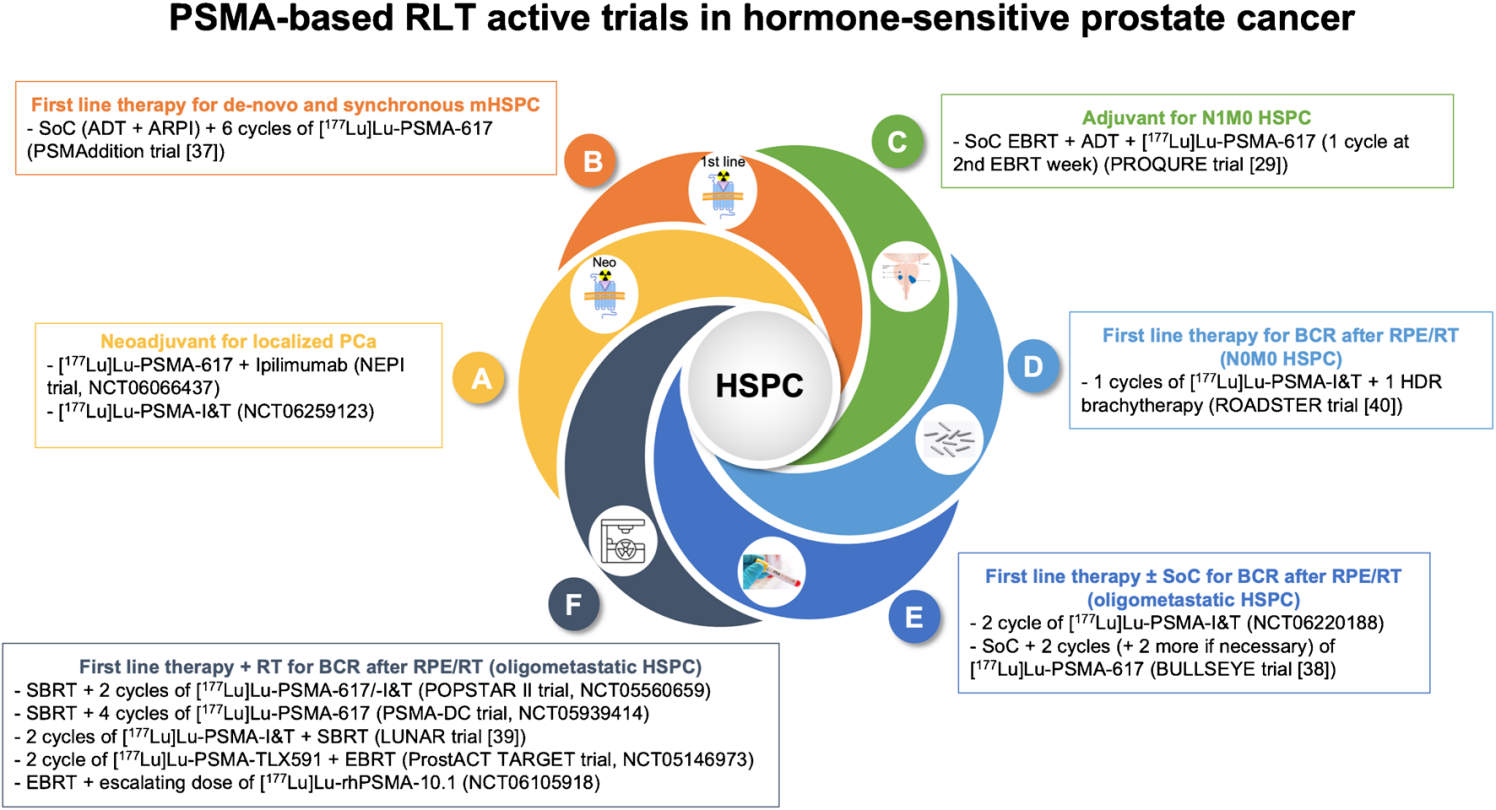
Main ongoing trials regarding PSMA-based RLT in HSPC

In Table 1 we resumed the main characteristics of the above-mentioned ongoing clinical trials using RLT in BCR HSPC. Figure 3 summarizes ongoing trials regarding PSMA-based RLT in HSPC treatment scenarios.

**Table 1:** Main characteristics of the described ongoing clinical trials using PSMA-based RL in HSPC

Study (Sponsor)	Scenario	Nation (mono/ multicenter)	Study participants	Enrollment Imaging	Study status (completion)	Study design	Primary endpoint(s)
NCT06220188 (Vienna Medical University)	BCR after RPE/RT	Austria (monocentre)	20	PSMA PET negative for local recurrence; distant mts allowed	Recruiting (01/2027)	Phase II, single arm, 2 cycles (3 + 6GBq) of [^177^Lu]Lu-PSMA-I&T at 6w intervals	PSA50, toxicity
ProstACT TARGET (Telix Pharmaceuticals) NCT05146973	Oligomts BCR after RPE/RT	Australia (multicentre)	50	PSMA PET negative for local recurrence; ≤ 5 pelvic PSMA PET positive lnd mts^*^	Active (08/2024)	Phase II, single arm, 2 cycles of 2.8GBq of [^177^Lu]Lu-DOTA-TLX591 at 2w intervals	PSA-FS
NCT06105918 (Emory University)	Locally recurrent after RPE	US (monocentre)	10	rhPSMA-7.3 PET/CT negative for distant metastases	Recruiting (04/2029)	Phase I, single-arm, escalating dose of consolidative [^177^Lu]Lu-rhPSMA-10.1 after rhPSMA-7.3 PET-guided EBRT	Safety, feasibility
PSMAddition (Novartis Pharmaceuticals) [37]	Untreated/minimally treated mHSPC	International (multicentre)	1145 (actual)	[^68^Ga]Ga-PSMA-11 positive disease	Ongoing (02/2026)	Phase III, randomized (1:1): Arm A: SoC (ADT + ARPI); Arm B: SoC + 6 cycles of 7.4GBq of [^177^Lu]Lu-PSMA-617 at 6w intervals	rPFS
BULLSEYE (Radboud University Medical Center) [38]	Oligomts BCR after RPE/RT	The Netherlands (monocentre)	58	≤ 5 bone/lnd lesions at [^18^F]PSMA-1007 PET/CT and wbMRI	Recruiting (01/2025)	Phase II, randomized (1:1): Arm A: SoC (watchful wait); Arm B: SoC + 2 cycles of 7.4GBq of [^177^Lu]Lu-PSMA-617 at 6w intervals + 2 more cycles if still PSMA-avid°°	PFS
POPSTAR II (Peter MacCallum Cancer Centre) NCT05560659	Oligomts BCR after RPE/RT	Australia (monocentre)	92	Positive [^68^Ga]Ga-PSMA-11 or [^18^F]DCFPyL PET/CT^**^	Recruiting (05/2026)	Phase II, randomized (1:1): Arm A: SBRT + 2 cycles of [^177^Lu]Lu-PSMA-617/I&T; Arm B: SBRT	PSA-FS
PSMA-DC (Novartis Pharmaceuticals) NCT05939414	Oligomts BCR after RPE/RT	International (multicentre)	450	Positive [^68^Ga]Ga-PSMA-11 or [^18^F]DCFPyL PET/CT^***^	Recruiting (06/2030)	Phase III, randomized (1:1): Arm A: SBRT + 4 cycles of 7.4GBq of [^177^Lu]Lu-PSMA-617 at 6w intervals; Arm B: SBRT	Time to ADT
LUNAR (Jonsson Comprehensive Cancer Center) [39]	Oligomts BCR after RPE/RT	US (monocentre)	100	≤ 5 asymptomatic lesions outside the prostate positive at [^68^Ga]Ga-PSMA-11 PET/CT°	Ongoing (09/2025)	Phase II, randomized (1:1): Arm A: 2 cycles of 6.8GBq of [^177^Lu]Lu-PSMA-I&T + SBRT°°°; Arm B: SBRT to PSMA-positive sites	PFS
ROADSTER (Glenn Bauman) [40]	Intra-prostatic-only recurrent after RT	Canada (monocentre)	Phase 1 = 12 Phase 2 = 30	Intra-prostatic-only recurrent PCa with PSMA PET/MRI SUV_max_ ≥3	Active (12/2024)	Phase I-II, randomized (1:1): Arm A: 1 cycle of 6.8GBq of [^177^Lu]Lu-PSMA-I&T + 1 HDR brachytherapy; Arm B: 2 HDR brachytherapy (both arms followed by re-biopsy)	Safety, feasibility

*ADT *androgen-deprivation therapy, *ARPI* androgen-receptor pathway inhibitor, *BCR* biochemical recurrence, *EBRT* external-beam radiation therapy, *FS* free-survival, HDR high-dose-rate, lnd lymph node, mts metastasis, *PET* positron emission tomography, PSA50 *PSA* reduction > 50%, *PSMA* prostate-specific membrane antigen, *RPE* radical prostatectomy, *rPFS* radiological progression-free survival, *RT* radiotherapy, *SBRT* stereotactic body radiation therapy, *SoC *standard of care, w week, wbMRI whole-body magnetic resonance imaging *at least one pelvic nodal lesion is ≥ 5 mm in the greatest dimension, SUVmax > 9 in enlarged nodes, and SUVmax > 3 in < 5 mm nodes **1–5 nodal/bone mts with E-PSMA RADS 4–5 (low/absent PSMA uptake is allowed) + at least 1 lesion with PSMA SUVmax twice of liver *** negative conventional imaging (CI, CT and bone scan) for M1 disease (CI can be positive only for local recurrence) with at least 1 PSMA-positive/CI-negative distant metastases with no liver/brain involvement °the smallest diameter ≥ 1 cm on CT or MRI or increased uptake on BS °°at interim [18F]PSMA-1007 PET/CT °°°dose-adapted on post-RLT PSMA PET

## Discussion

Following the FDA and EMA approval in 2022, PSMA RLT has been rapidly integrated into PCa management guidelines and is increasingly adopted worldwide [4]. While other molecules are still under evaluation for PCa theranostics [41, 42], PSMA RLT is already indicated for mCRPC patients exhibiting one or more metastatic lesions that show high PSMA PET expression (above half of the parotid uptake). Eligible patients must have progressed on ARPI and taxane, and must not have relevant PSMA PET-negative metastases or active/lytic bone lesions [4].

The observed improvement in OS, coupled with a PSA reduction and a favorable safety profile in the post-taxane CRPC setting has prompted the consideration of [^177^Lu]Lu-PSMA RLT earlier in the treatment sequence, in both pre-taxane CRPC and HSPC settings. Upcoming results from the SPLASH [43], ECLIPSE trials (NCT05204927) and long-term results from the ENZAP study [44] are anticipated to further validate the efficacy of this treatment in CRPC patients before docetaxel or cabazitaxel therapy.

ADT, ARPI and chemotherapy may determine toxicities and adverse events: therefore, patients’ comorbidities, performance status, ability to tolerate the treatment AEs, and affordability need to be considered in clinical practice when deciding on the appropriate course of treatment. Despite limited data, early RLT in HSPC has shown preliminary encouraging results, representing a valid alternative for selected patients who refuse or are unable to receive the SoC, potentially delaying the onset of castration-resistant status and the need for other therapies.

Preliminary data [32, 33, 35, 36] showed that the early use of RLT in HSPC is safe with reported grade 3–4 toxicity in < 5% versus ~ 50% on later-stage PCa [7, 9] due to better organ reserve (i.e., bone marrow). Interestingly, early RLT also showed similar dosimetry compared with high-volume mCRPC, confirming that the “sink effect” is of less concern in this setting [33]. Regarding efficacy, and with all due limitations, RLT in HSPC demonstrated a more significant PSA reduction and longer PFS (~ 12 m vs. ~ 9 m) than in mCRPC (~ 65% vs. ~ 50%). This is likely due to a more differentiated and lower tumor burden mainly limited to lymph node lesions, in which RLT is more effective [7, 9, 32, 33, 35]. Indeed, introducing [^177^Lu]Lu-PSMA RLT at an earlier stage could potentially target a more homogenous PSMA-expressing disease, leading to a more robust response compared to the heterogeneous PSMA expression often observed in the CRPC setting [45]. Despite limited data on PSMA expression in hormone-sensitive disease [46], it is known that PSMA is involved in the PI3K–mTOR signaling pathway, with an increased expression on androgen blockade in clonal subpopulations of mCRPC [47]. However, results from the ENZA-P study have shown benefits in terms of PFS, PSA response and pain reduction of a synergic approach using I line [^177^Lu]Lu-PSMA RLT 2 and 8 weeks after the start of enzalutamide in mCRPC with high-risk characteristics for early enzalutamide failure [44]. In this setting, preclinical data suggested that pre-treatment with enzalutamide before RLT results in more substantial DNA damage and significantly upregulated PSMA surface levels in PCa cell-line (LNCaP) [48]. Moreover, the uptake and internalization of [^177^Lu]Lu-PSMA-617 enhanced considerably in LNCaP cells following enzalutamide treatment [49]. Indeed, another critical aspect to be considered is the hormone-therapy influence on PSMA expression which can potentially affect not only PSMA imaging but, also, PSMA-based RLT efficacy. Despite disease heterogeneity and discordant data in the literature, it can be assumed that PSMA expression can be increased within 4 weeks after the hormone therapy start (more evidence in the CRPC status), then showing a gradual reduction [18, 50]. Waiting for more robust data (NCT05919329), inherent considerations must be, therefore, done for PSMA-based RLT studies including PSMA imaging timing, especially if any association/sequencing with hormone therapy is foreseen. Indeed, short-term hormone therapy can potentially create a window for enhanced PSMA-imaging sensitivity [18] and PSMA-based RLT effectiveness [44].

Given the favourable safety profile of [^177^Lu]Lu-PSMA RLT and the ability to repeatedly assess PSMA expression via SPECT and PET, this treatment could theoretically be applied in the HSPC space and, if necessary, repeated when the disease becomes castration-resistant. Initial data have confirmed the efficacy and safety of extended/rechallenge [^177^Lu]Lu-PSMA RLT in mCRPC [49], though more data are needed (NCT06288113).

Alpha radiation [52] has a shorter penetration range in human tissue (< 0.1 mm versus up to 2 mm for beta minus) and higher energy (4–9 MeV versus up to 2.3 MeV for beta minus), primarily inducing DNA double-strand breaks as opposed to the mainly single-strand breaks caused by beta minus radiation [5, 53, 54]. Based on this, combining PSMA ligand with beta- (e.g., [^177^Lu]Lu-PSMA) and alpha-emitting (e.g., [^225^Ac]Ac-PSMA) may improve treatment efficacy compared to using only beta-emitting agents [52]. However, although evidence is still limited, this combination may also result in higher toxicities [35] than the use of de-escalating [^225^Ac]Ac-PSMA-alone dosage [28]. Early-phase HSPC, where patients usually have better organ reserve, may therefore be more suitable for testing this combination than the later phases of the disease (NCT06229366). Further promising nuclides are under evaluation, such as [^212^Pb]Pb-PSMA which is an alpha emitter enabling post-injection biodistribution imaging [55], and [^161^Tb]Tb-PSMA a beta minus nuclide (with higher energy than [^177^Lu]Lu) able to co-emit Auger particles and conversion electrons theoretically resulting in disseminated higher tumor-absorbed doses [56]; however, until now published data and listed ongoing trials are limited to the CRPC stage of disease.

An interesting and more studied approach is the combination of PSMA RLT with RT, where RLT can treat even micrometastasis, also acting as radiosensitiser [57], while RT is more effective towards assessable lesions [58]. This combination allows therapeutic absorbed dose intensification while limiting OAR exposure [36].

Even in the neoadjuvant setting, waiting for ongoing trial results, preliminary data demonstrated that RLT followed by RPE is safe, leads to substantial radiation doses to the primary tumors (median 35 Gy) and lymph nodes and does not compromise surgical safety [23, 24].

Before reflecting on how to potentially integrate PSMA-RLT early into existing treatment protocols, we must wait/demonstrate clinically meaningful results/improvements compared to the SoC. A factor that must be considered is the disease heterogeneity which determines different survival and response to RLT [59]. Indeed, therapeutical combination with PSMA-based RLT can be more appropriate/effective in case of disease heterogeneity; the choice on how to select the best concomitant agent is far from being demonstrated, however, it can, at least, partially rely on disease burden (low- or high-volume), stage (HSPC or CRPC), patients characteristics and, of course, availability. Further consideration could be done regarding the optimal RLT timing, number of cycles and dosage; finally, there will certainly be room also for alpha-emitters [52–56]), association with immunotherapy [NCT06388369, 60] and new molecules [NCT05146973, NCT06066437]. With the potential for increased efficacy, the use of combination therapies will need to be balanced against possibly increased safety issues.

Despite the actual paucity of data, the main ongoing/recruiting trials indicate a growing interest in earlier RLT applications, reflecting a natural shift in the theranostics paradigm. Indeed, PSMA RLT has the potential to deliver an early cytotoxic payload to disseminated PSMA-expressing microscopic disease below the resolution of modern scanners, potentially reducing rates of systemic progression, which is the most frequent pattern of relapse in this setting. RLT may also induce an immunogenic effect by changing and stimulating the tumor microenvironment, enhancing tumor cell recognition by the immune system. This could effectively provide a patient-specific tumor vaccine, improving the systemic response and potential survival, moving beyond the concept of “treat what you see”, towards “treat what you see but also what you do not see”.

## Conclusion

Clinically, PSMA RLT is an effective option for PSMA PET-positive mCRPC patients who have progressed on ARPI and taxane treatments, representing a potential alternative before the actual indication in highly selected patients unfit to conventional therapies. Indeed, emerging data suggest that PSMA RLT holds significant potential in PCa patients with earlier disease, from the neoadjuvant to the BCR setting. In these stages, the lower tumor burden, more frequent exclusive nodal involvement, and higher organ reserve may improve treatment efficacy and allow for treatment combinations (e.g., with RT, target therapies or by adding alpha- to beta-emitting radiopharmaceuticals) while maintaining a less toxic profile.

## Data Availability

This is a literature review and therefore no datasets were generated.
